# Safety and efficacy of laxatives after major abdominal surgery: systematic review and meta‐analysis

**DOI:** 10.1002/bjs5.50301

**Published:** 2020-05-27

**Authors:** N. N. Dudi‐Venkata, W. Seow, H. M. Kroon, S. Bedrikovetski, J. W. Moore, M. L. Thomas, T. Sammour

**Affiliations:** ^1^ Colorectal Unit, Department of Surgery, Royal Adelaide Hospital Adelaide South Australia Australia; ^2^ Discipline of Surgery, Faculty of Health and Medical Sciences School of Medicine, University of Adelaide Adelaide South Australia Australia

## Abstract

**Background:**

Recovery of gastrointestinal function is often delayed after major abdominal surgery, leading to postoperative ileus (POI). Enhanced recovery protocols recommend laxatives to reduce the duration of POI, but evidence is unclear. This systematic review aimed to assess the safety and efficacy of laxative use after major abdominal surgery.

**Methods:**

Ovid MEDLINE, Embase, Cochrane Library and PubMed databases were searched from inception to May 2019 to identify eligible RCTs focused on elective open or minimally invasive major abdominal surgery. The primary outcome was time taken to passage of stool. Secondary outcomes were time taken to tolerance of diet, time taken to flatus, length of hospital stay, postoperative complications and readmission to hospital.

**Results:**

Five RCTs with a total of 416 patients were included. Laxatives reduced the time to passage of stool (mean difference (MD) −0·83 (95 per cent c.i. −1·39 to −0·26) days; *P* = 0·004), but there was significant heterogeneity between studies for this outcome measure. There was no difference in time to passage of flatus (MD −0·17 (−0·59 to 0·25) days; *P* = 0·432), time to tolerance of diet (MD −0·01 (−0·12 to 0·10) days; *P* = 0·865) or length of hospital stay (MD 0·01(−1·36 to 1·38) days; *P* = 0·992). There were insufficient data available on postoperative complications for meta‐analysis.

**Conclusion:**

Routine postoperative laxative use after major abdominal surgery may result in earlier passage of stool but does not influence other postoperative recovery parameters. Better data are required for postoperative complications and validated outcome measures.

## Graphical abstract

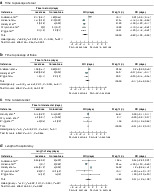

## Abstract (es)

**Antecedentes:**

El retraso en la recuperación de la función gastrointestinal después de una cirugía abdominal mayor es frecuente y conlleva la aparición de un íleo postoperatorio (*postoperative ileus*, POI). Los protocolos de rehabilitación multimodal recomiendan la utilización de laxantes para reducir la duración del POI, pero su evidencia no es concluyente. Esta revisión sistemática tuvo como objetivo evaluar la seguridad y la eficacia de la utilización de laxantes tras cirugía abdominal mayor.

**Métodos:**

Se realizó una búsqueda de los estudio clínicos aleatorizados (*randomised controlled trials*, RCTs) centrados en la cirugía abdominal mayor electiva, abierta o mínimamente invasiva, en las bases de datos Ovid MEDLINE, EMBASe, Cochrane Library y PubMed, desde el inicio hasta mayo de 2019. La variable principal fue el tiempo transcurrido hasta la primera deposición. Las variables secundarias fueron el tiempo hasta tolerar la dieta, el tiempo hasta la emisión de ventosidades, la duración de la estancia hospitalaria, las complicaciones postoperatorias y los reingresos hospitalarios.

**Resultados:**

Se incluyeron cinco RCTs con un total de 416 pacientes. Los laxantes redujeron el tiempo hasta la primera deposición (diferencia media, *mean difference*, MD − 0,83 (i.c. del 95% −1,39 a −0,26) días; *P* = 0,004) pero hubo una heterogeneidad significativa entre los estudios para la medida de este resultado. No hubo diferencias en el tiempo hasta la emisión de ventosidades (DM − 0,17 (−0,59 a 0,25) días; *P* = 0,432), tiempo hasta la tolerancia de la dieta (DM − 0,01 (−0,12 a 0,10) días; *P* = 0,865) y la duración de la estancia hospitalaria (DM 0,01 (−1,36 a 1,38) días; *P* = 0,992). No había datos suficientes de las complicaciones postoperatorias para efectuar un metaanálisis.

**Conclusión:**

El uso rutinario de laxantes en el postoperatorio de una cirugía abdominal mayor puede acelerar el tránsito de heces, pero no influye en otras variables de la recuperación postoperatoria. Se requieren datos de mayor calidad para evaluar las complicaciones postoperatorias y medidas de resultados validadas.

## Introduction

Recovery of gastrointestinal function is often delayed after major abdominal surgery, leading to postoperative ileus (POI)^1^. For patients experiencing POI, it is a source of significant morbidity and discomfort, causing vomiting, abdominal distension and intolerance to diet, and often leading to invasive interventions such as insertion of a nasogastric tube^2^, ^3^. Postoperative complications such as POI can have a significant impact on patient outcome, in terms of short‐term recovery, long‐term survival, and quality of life^4^, ^5^, ^6^. Healthcare expenditure is almost twice as high when patients develop POI compared with that for patients who do not^7^, ^8^, ^9^, and there is evidence that 91 per cent of these increased costs relate directly to the patient's immediate postoperative stay^7^.

Since the implementation of enhanced recovery protocols (ERPs), the management of patients undergoing abdominal surgery has improved, with a reduction in the incidence of complications^10^, ^11^, ^12^, ^13^, ^14^, ^15^, ^16^. However, despite the widespread adoption of ERPs, the incidence of POI remains high at around 10–30 per cent, with delayed return of gastrointestinal function continuing to be a common barrier to discharge from hospital^4^, ^17^, ^18^, ^19^. One possible reason for this could be because of a complex, intricate, and as yet incompletely defined relationship between the neuroinflammatory, vagal and drug‐induced processes underlying the pathophysiology of POI^20^, ^21^. Multimodal strategies have been employed to improve the return of gastrointestinal function after surgery, including the routine use of postoperative laxatives^22^, ^23^. The recommendations for laxative use are varied in different international ERP protocols, with only weak evidence quoted to support their efficacy^24^. In addition, published data on the safety of postoperative laxatives in this setting are limited, in particular with regard to anastomotic leak.

This systematic review aimed to assess the safety and efficacy of laxative use after major abdominal surgery.

## Methods

The study protocol was registered prospectively with the PROSPERO database of systematic reviews (CRD42019126282). PRISMA guidelines^25^ were used for conducting and reporting the results of this study.

### Search strategy

Two independent reviewers performed a systematic search of the MEDLINE (1946 to 21 May 2019), Embase (1974 to 21 May 2019), Cumulative Index to Nursing and Allied Health Literature (CINAHL) (EBSCOhost) databases (1974 to 21 May 2019), and the Cochrane Database of Systematic Reviews, Cochrane Clinical Trials Register, and Database of Abstracts on Reviews and Effectiveness. All ‘Primary Registries’ listed in the WHO Registry Network (including ClinicalTrials.gov) were searched for ongoing (unpublished) RCTs (searched on 22 May 2019). The detailed search strategy is shown in *Table S1* (supporting information). Medical subject headings (MeSH) and keyword search terms related to ‘ERAS’, ‘recommendations’, ‘laxatives’, ‘abdominal’, ‘surgery’, ‘prevention’, ‘postoperative’, ‘ileus’ and ‘gastrointestinal 2’ (GI‐2) were used. Unpublished data were also sought from authors of trials listed in the registry. The search was limited to studies published in the English language. The last search update was on 22 May 2019.

### Eligibility criteria

Studies were included if they were RCTs conducted in patients aged more than 16 years undergoing elective open or minimally invasive major abdominal surgery, and specifically assessed the effect of laxatives on the return of gastrointestinal function, defined by time to passage of stool or using a validated measure such as GI‐2 or GI‐3. GI‐2 is a composite measure of tolerance to solid diet for 24 h (no vomiting) and passage of stool, whereas GI‐3 is a composite measure of tolerance to solid diet for 24 h (no vomiting) and passage of flatus^26^. Eligible articles with a description of interventions directed towards stimulation of bowel motility, prevention of POI or reducing its duration, or facilitating the return of gastrointestinal function after surgery were included in the final analysis. All gastrointestinal (colorectal, gastric, small bowel, hepatic, pancreatic resection), urological (nephrectomy, cystectomy, prostatectomy) and gynaecological (uterus and ovary resection, pelvic floor reconstruction) operations, undertaken for any indication, were considered as major abdominal surgery. Studies with quasirandomized, prospective and retrospective design, and case–control studies were excluded.

### Study selection

All identified titles and abstracts were reviewed independently by two investigators. This was followed by a further review of the full texts of potentially relevant studies. Bibliographies of relevant articles also underwent a manual cross‐reference search to identify any other studies that had been missed in the search. Any potential differences over study selection were resolved by consensus and, if needed, adjudication was undertaken by a third reviewer.

### Data collection process

Data of all included studies were extracted independently by two reviewers using a standard data extraction form. Outcome measures data, including GI‐2 or GI‐3, time taken to passage of first stool, time taken to tolerance of solid food, time taken to first flatus, length of hospital stay, postoperative complications, adverse drug effects and readmission to hospital, were extracted. In addition to the measured outcomes, other data related to general study characteristics, including author name, country of origin, year of study, study type, patient population, number of patients in control and intervention arm, site of surgery, type of intervention (laxatives) and route of administration, were also extracted. All the data were cross‐checked at the end, and any discrepancies in the extraction of the data were resolved by the third reviewer.

### Risk of bias in individual studies

The Cochrane Collaboration risk‐of‐bias tool^27^ was used independently by two authors to assess the methodological quality of individual RCTs. A consensus was sought for any disagreements.

### Assessment of quality of evidence

Quality of evidence and summary of findings were tabulated using the GRADEpro Guideline Development Tool (McMaster University, Hamilton, Ontario, Canada).

### Statistical analysis

JBI SUMARI online software (https://www.jbisumari.org/) was used for quantitative analysis of aggregated data. An intention‐to‐treat methodology was adopted. Mean estimates were calculated by the method proposed by Wan *et al*.^28^. Effect estimates are reported as odds ratios (ORs) and weighted mean differences (MDs) with 95 per cent c.i. for dichotomous and continuous outcomes respectively. Considering the heterogeneity between the studies, pooled estimates of effect were calculated using a random‐effects model. Statistical significance was set at *P* < 0·050 for the degree of heterogeneity, which was determined by the χ^2^ test. Heterogeneity measured by the *I* ^2^ statistic was perceived as considerable when the *I* ^2^ value was above 75 per cent^29^.

To decrease potential bias introduced by diverse indications and surgical methods, a planned subgroup analysis was performed on primary and secondary outcomes for patients who underwent colorectal surgery.

## Results

The literature search identified 323 studies. After removal of 13 duplicates, a further 280 studies were excluded after screening by title and abstract. Four authors of unpublished studies from trial registries were contacted via e‐mail to enquire about the progress of their study and the relevant results, if any. One author responded and confirmed that the trial was not yet completed. In addition, authors of all the RCTs included in the meta‐analysis were contacted, requesting raw trial data to explore finer details of the trial further, but none responded.

**Figure 1:**
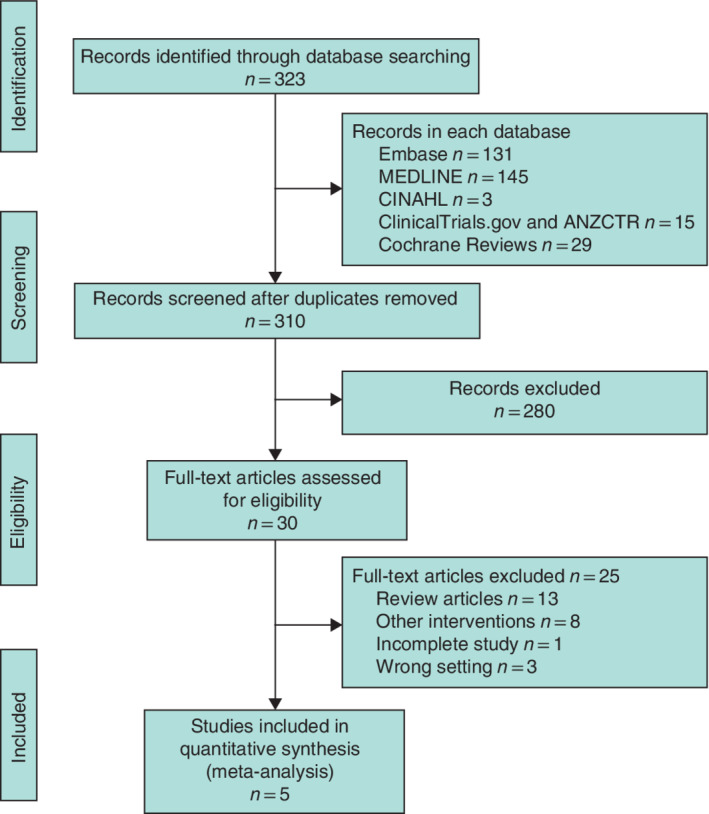
PRISMA diagram for the review CINAHL, Cumulative Index to Nursing and Allied Health Literature; ANZCTR, Australian New Zealand Clinical Trials Registry.

Thirty studies met the prespecified inclusion criteria and were evaluated by full‐text analysis. This analysis initially yielded six RCTs for inclusion, but one^30^ of these was excluded on further review as participants in this study received baseline laxative (docusate sodium) in both intervention and control groups. Hence, five RCTs^31^, ^32^, ^33^, ^34^, ^35^ were finally selected for inclusion (*Fig*. 1).

### Characteristics of included studies

The five included RCTs^31^, ^32^, ^33^, ^34^, ^35^ were published between 2007 and 2011, and included 416 patients (209 (range 10–100) in the laxative group and 207 (10–100) in the placebo group). Study characteristics, interventions and outcomes are summarized in *Tables* 1 and 2. The studies were conducted in six countries. Three RCTs^31^, ^34^, ^35^ involved patients with colorectal disease, and the other two were in patients undergoing hysterectomy^32^ and hepatic resection^33^.

**Table 1:** Characteristics of the included studies

				No. of patients				
Reference	Country	Year	Patient population	Intervention	Control	Total	Intervention	Route of administration
Andersen *et al*.^31^	Denmark	2012	Colorectal	31	31	62	Magnesium oxide	Oral
Hansen *et al*.^32^	Denmark	2007	Hysterectomy	34	32	66	Magnesium oxide	Oral
							Disodium phosphate	
Hendry *et al*.^33^	UK	2010	Hepatic resection	34	34	68	Magnesium oxide	Oral
	Netherlands							
Wiriyakosol *et al*.^34^	Thailand	2007	Colorectal	10	10	20	Bisacodyl	Rectal
Zingg *et al*.^35^	Switzerland	2008	Colorectal	100	100	200	Bisacodyl	Oral

**Table 2:** Characteristics of study interventions and outcomes

			Outcomes	
Reference	Intervention	Control	Primary	Secondary
Andersen *et al*.^31^	Magnesium oxide 1 g orally, twice daily D0 at 18.00 hours D1–7 twice daily	Placebo	Time to first defaecation (h) Time to passage of flatus (h) Cumulative median no. of orally consumed drinks, supplementary protein drinks and solid foods on D0, D2–3	LOS (days)
Hansen *et al*.^32^	Magnesium oxide 1 g Disodium phosphate: 15 ml D0 6 h after surgery D1 twice daily	Placebo	Time to first defaecation (h) Pain score	
Hendry *et al*.^33^	Magnesium oxide 1 g twice daily D0 to day of discharge	No placebo Standard of care	Time to first defaecation (days) Time to passage of flatus (days)	Oral nutritional intake D1–3 LOS (days)
Wiriyakosol *et al*.^34^	Bisacodyl suppositories 10 mg once daily to twice daily D3 If no defaecation after first dose, second dose administered 12 h later	Placebo	Time to first defaecation (days)	Time to passage of flatus (days) Time to tolerance of diet (days) LOS (days)
Zingg *et al*.^35^	Bisacodyl 10 mg orally, twice daily D −1 to D3	Placebo	Time to first defaecation (days) Time to passage of flatus (days) Time to tolerance of solid diet (days)	LOS (days)

LOS, length of hospital stay; D0, day of surgery; D1, day 1 after surgery; D −1, 1 day before surgery.

### Cochrane Collaboration risk‐of‐bias assessment

All seven components were assessed as low risk for three RCTs^33^, ^34^, ^35^, but there was a high risk of attrition bias for two trials^31^, ^32^ owing to incomplete outcome data. Only a small number of trials were included in this study, so a funnel plot for potential publication bias was not generated as this would not have enough data points to be meaningful. Summary graphs for risk of bias are shown in *Figs* *S1* and *S2* (supporting information).

### Study interventions

The most tested laxatives were magnesium oxide and bisacodyl. Oral magnesium oxide was the intervention used in two studies^31^, ^33^, bisacodyl in two studies (one per rectum^34^ and one orally^35^), and disodium phosphate along with magnesium oxide in one study^32^ (*Table* 1). Laxative regimens differed in terms of the day of starting the first dose and the total duration. Three studies^31^, ^32^, ^33^ administered the laxatives from the day of surgery (D0), one^35^ from the day before surgery (D −1), and one^34^ from day three after surgery (D3). Four studies^31^, ^32^, ^34^, ^35^ were double‐blinded with placebo used as the control, whereas one study^33^ was open‐labelled to the interventions used because of lack of a feasible placebo and used standard of care in the control arm (no placebo).

### Quantitative analysis

#### Return of gastrointestinal function

**Figure 2:**
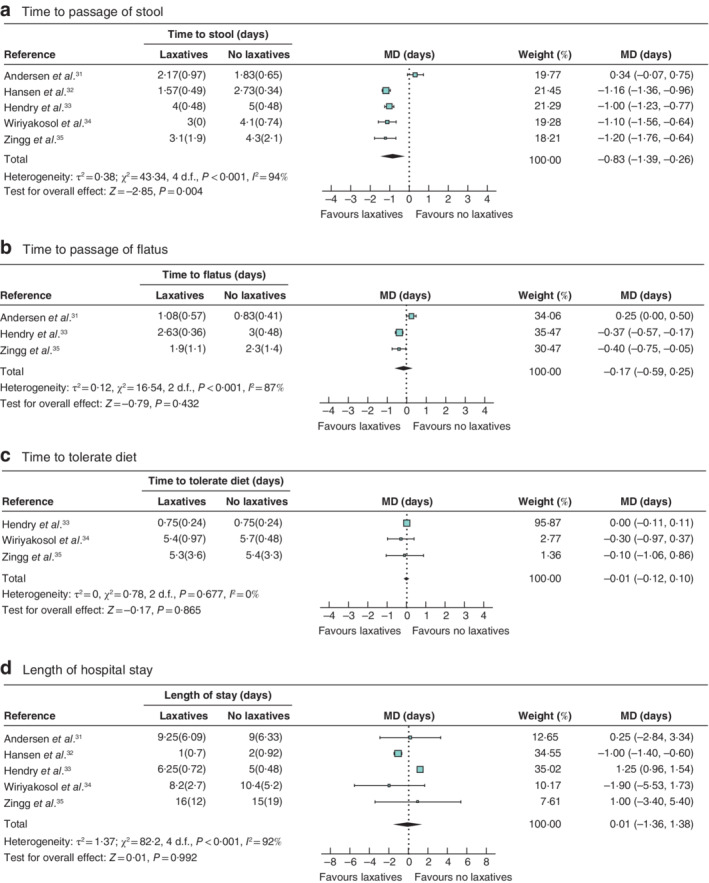
Forest plots comparing time to passage of stool and flatus, time to tolerate diet and length of hospital stay in laxatives and control groups, all studies **a** Time taken to passage of stool; **b** time taken to passage of flatus; **c** time to tolerate diet; **d** length of stay in hospital. Values in parentheses are 95 per cent c.i. unless indicated otherwise; *values are mean(s.d.). An inverse‐variance random‐effects model was used for meta‐analysis. Weighted mean differences (MDs) are shown with 95 per cent confidence intervals.

None of the studies reported GI‐2. One study^35^ reported significantly shorter time to GI‐3 in the laxative *versus* control group (median 3 (range 1–12·3) *versus* 3·7 (1·7–10·7) days respectively; *P* = 0·007). Time to the passage of stool was reported by all five studies^31^, ^32^, ^33^, ^34^, ^35^; there was a statistically significant difference for laxatives compared with control (MD −0·83 (95 per cent c.i. −1·39 to −0·26) days; *P* = 0·004), although there was significant heterogeneity between the studies for this outcome (*I* ^2^ = 94 per cent; *P* < 0·001) (*Fig*. 2 *a*).

#### Time to passage of flatus

Time to passage of flatus was reported by three studies^31^, ^33^, ^35^; there was no statistically significant difference between laxatives and control (MD −0·17 (95 per cent c.i. −0·59 to 0·25) days; *P* = 0·432). There was significant heterogeneity between the studies for this outcome (*I* ^2^ = 87 per cent; *P* < 0·001) (*Fig*. 2 *b*).

#### Time to a tolerance of diet

Three studies^33^, ^34^, ^35^ reported time to a tolerance of diet; there was no significant difference between the laxatives and control group (MD −0·01 (95 per cent c.i. −0·12 to 0·10) days; *P* = 0·865). There was no significant heterogeneity between the studies for this outcome (*I* ^2^ = 0 per cent; *P* = 0·677) (*Fig*. 2 *c*).

#### Length of hospital stay

Length of hospital stay was reported by all studies^31^, ^32^, ^33^, ^34^, ^35^; there was no significant difference between the laxatives and control group (MD 0·01 (95 per cent c.i. −1·36 to 1·38) days; *P* = 0·992). There was significant heterogeneity between the studies for this outcome (*I* ^2^ = 92 per cent; *P* < 0·001) (*Fig*. 2 *d*).

#### Postoperative complications

Pooled analysis was not done on these outcomes as the data available in the included studies were limited. Superficial surgical‐site infections were reported in two (20 per cent) of ten patients in the control group in one study^34^, whereas another^31^ reported one death (3 per cent) of 31 patients in the laxative group from cardiac arrest on the second postoperative day; however, the complication profile was similar in both groups in other reports^32^, ^33^. No significant difference in surgical complications (anastomotic leak, surgical‐site infection, abdominal fascia dehiscence, postoperative bleeding) or non‐surgical morbidity (pneumonia, cardiac failure, pulmonary embolism, renal failure, urinary tract infection) was reported in another RCT^35^; overall surgical morbidity in this study was 23·1 per cent, whereas non‐surgical complications occurred in 13 per cent of all patients.

#### Subgroup analysis

**Figure 3:**
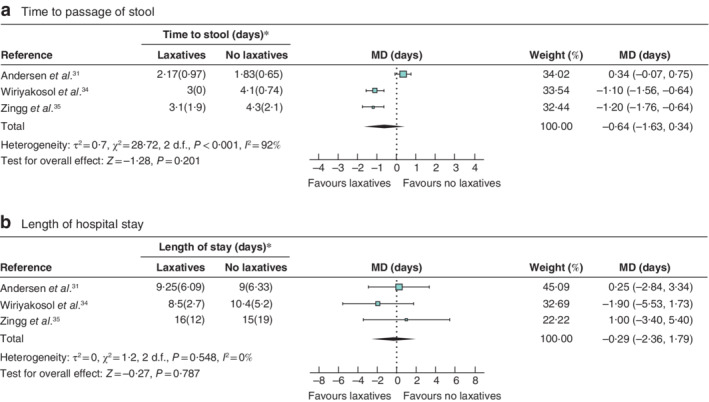
Forest plots comparing time to passage of stool and length of hospital stay in laxatives and control groups, colorectal studies only **a** Time taken to passage of stool; **b** length of stay in hospital. Values in parentheses are 95 per cent c.i. unless indicated otherwise; *values are mean(s.d.). An inverse‐variance random‐effects model was used for meta‐analysis. Weighted mean differences (MDs) are shown with 95 per cent confidence intervals.

In the three studies^31^, ^34^, ^35^ with only colorectal patients, no significant difference was found in time to passage of stool between the laxatives and control group (MD −0·64 (95 per cent c.i. −1·63 to 0·34) days; *P* = 0·201). Heterogeneity was significant for this outcome (*I* ^2^ = 92 per cent; *P* < 0·001) (*Fig*. 3 *a*). Length of hospital stay in the laxatives and control group showed no significant difference (MD −0·29 (−2·36 to 1·79) days; *P* = 0·787). There was no heterogeneity between studies for this outcome (*I* ^2^ = 0 per cent; *P* = 0·548) (*Fig*. 3 *b*). Anastomotic leak rates for these studies ranged between 0 per cent in both groups^34^, 8·3 *versus* 6·3 per cent (*P* > 0·99)^31^, and 8·4 *versus* 4·7 per cent (*P* = 0·365)^35^ in the laxative *versus* control group respectively.

### GRADE assessment for quality of evidence

Using the Grading of Recommendations, Assessment, Development and Evaluations (GRADE) criteria, the overall quality of evidence for time taken to defaecation and to tolerate diet was low, whereas it was very low for time taken to pass flatus and length of hospital stay (*Table S2*, supporting information).

## Discussion

This meta‐analysis of all available RCTs evaluating laxatives after major abdominal surgery demonstrates that time to passage of stool is shorter with laxative use. However, there was significant heterogeneity between studies for this outcome measure, and time to passage of flatus, time to tolerance of diet and length of stay in hospital were unaffected. There were insufficient data to draw conclusions on the safety profile of laxatives used in this setting.

The effect of laxatives on bowel motility depends on the type and mechanism of action. Some laxatives (such as sennosides) work by stimulating gut activity and others (such as polyethylene glycol) by osmotic distension of the bowel lumen. A distended colon is more likely to initiate colonic contractions leading to a bowel movement than a non‐distended, empty colon^36^, ^37^. There is also evidence of a postoperative ‘brake’ system in or around the rectosigmoid colon that acts as a physiological sphincter by causing retrograde contractions and inhibiting normal passage of enteric contents^38^. It is plausible that giving laxatives per rectum could counteract this pathophysiological effect, initiating an antegrade bowel movement. Published evidence of the use of laxatives to improve gastrointestinal function after surgery dates back to the late 1990s^39^, ^40^, ^41^, ^42^. ERP guidelines commonly recommend using laxatives to stimulate bowel motility after surgery; however, these recommendations are not uniform and vary geographically. Moreover, the evidence behind these recommendations is quoted to be weak in several guidelines^24^.

The data in this review suggest there may be a benefit of laxatives after abdominal surgery. However, although earlier passage of stool is typically associated with earlier recovery of gastrointestinal function, interpretation is limited when measured in isolation. Most validated measures of gastrointestinal recovery are composite scores including other relevant parameters^23^. It is interesting that there was no difference in time to tolerance of diet or time to discharge in the present meta‐analysis. This could mean that laxatives result in a stimulated bowel movement, but not in improved recovery after surgery. Another potential explanation could be that, although well patients in the intervention arm passed stool earlier overall, there was no difference in rates of POI in the subset of patients, leading to a similar, longer, hospital stay overall in both groups. As rates of POI and validated outcome measures such as GI‐2 were not recorded in most studies, it is not possible to assess this further.

This review has several limitations, including the significant heterogeneity of the included studies, lack of validated outcome measures, small sample sizes, and variation in the types of operation performed and types of intervention used. In addition, data on complications were not adequate for meta‐analysis.

Given that most common laxatives are cheap medications with a favourable toxicity profile, their use deserves further exploration in the postoperative setting^21^. To this end, further higher‐powered RCTs are required with a specific focus on validated measures of gastrointestinal recovery and accurate collection of complications data (such as anastomotic leak). It remains unclear whether laxatives should be used after surgery in selected patients after abdominal surgery, or as a routine in all postoperative patients. The type and dose of laxative also varied between the included studies, and there may be a role for using a combination of laxatives with different mechanisms of action (both direct activity of bowel function and osmotic distension), as this is common in other aspects of enhanced recovery protocols (for example, multimodal pre‐emptive analgesia and antiemetics). The STIMULAX RCT (Australian New Zealand Clinical Trials Registry number ACTRN12618001261202), which is currently recruiting patients undergoing colorectal surgery, should address some of these questions.

## Supporting information


**Appendix S1:** Supporting information
