# Supplementary material for: Safety and efficacy of laxatives after major abdominal surgery: systematic review and meta‐analysis
Source: BJS Open. 2020 May 27;4(4):577–86. doi: 10.1002/bjs5.50301 (PMC7397346; doi:10.1002/bjs5.50301)

**BJS5_50301**

**Safety and efficacy of laxatives after major abdominal surgery: systematic review and meta-analysis**

**N. N. Dudi-Venkata, W. Seow, H. M. Kroon, S. Bedrikovetski, J. W. Moore, M. L. Thomas and T. Sammour**

**Table S1** Search strategy

| Database: Ovid MEDLINE® Epub Ahead of Print, In-Process & Other Non-Indexed Citations, Ovid MEDLINE® Daily and Ovid MEDLINE® < 1974 to 21^st^ May 2019>, Embase <1974 to 21^st^ May 2019>, CINAHL with full text (EBSCOhost) <1974 to 21^st^ May 2019>, EBM Reviews – Cochrane Database of Systematic Reviews, Clinical Trials Register, Database of Abstracts on Reviews and Effectiveness <22^nd^ May 2019>, World Health Organisation (WHO) Registry Network (including Clinical Trials.gov) and ANZCTR <22^nd^ May 2019>  [mp= ti, ot, ab, sh, hw, kw, tx, ct, tn, dm, mf, dv, fx, dq, nm, kf, px, rx, an, ui, sy] | |
| --- | --- |
| ERAS | 1. ERAS.mp 2. Fast track Protocols.mp 3. Multimodal pathway.mp 4. Multimodal rehabilitation.mp 5. Or/ 1-4 |
| Recommendations | 1. Recommendations.mp 2. Guidelines.mp 3. Protocols.mp 4. Or/ 6 - 8 |
| Laxatives | 1. Laxatives.mp 2. Aperients.mp 3. Cathartic.mp 4. Or/ 10 - 12 |
| Abdominal | 1. Abdominal.mp 2. Gastrointestinal.mp 3. Colon.mp 4. Rectum.mp 5. Or/ 14 - 17 |
| Surgery | 1. Abdominal surgery.mp 2. Digestive System Surgical Procedure.mp 3. Digestive surgery.mp 4. Colorectal surgery.mp 5. Colectomy.mp 6. General surgery.mp 7. Surgical oncology.mp 8. Gynaecologic surgery.mp 9. Laparoscopy.mp 10. Laparotomy.mp 11. Or/ 19 - 28 |
| Prevention | 1. Prevention.mp 2. Primary prevention.mp 3. Secondary prevention.mp 4. Or/ 30 - 32 |
| Postoperative | 1. Postoperative.mp 2. Postoperative period.mp 3. Postoperative care.mp 4. Post anaesthetic care.mp 5. Postoperative complications.mp 6. Or/ 34 - 38 |
| Ileus | 1. Ileus.mp 2. Paralytic ileus.mp 3. Postoperative ileus.mp 4. Gastrointestinal motility.mp 5. Gastrointestinal tract function.mp 6. Bowel function.mp 7. Gastrointestinal recovery.mp 8. Digestive system recovery.mp 9. Intestinal pseudo-obstruction.mp 10. Colonic pseudo-obstruction.mp 11. Postoperative Complications.mp 12. Or/ 40 - 50 |
| Gastrointestinal 2 (GI-2 is a composite measure of tolerance to solid diet for 24 hrs (no vomiting) AND passage of stool) | 1. Gastrointestinal 2.mp 2. GI-2.mp 3. Time to tolerance of solid food.mp 4. Time taken tolerance of diet.mp 5. Time to passage of first stool.mp 6. Time to passage of stool.mp 7. Return of bowel function.mp 8. Return of gastrointestinal function.mp 9. Or/ 52 - 59 |
|  | 1. AND/ 5, 9, 13, 18, 29, 33, 39, 51, 60 |

**Table S2** GRADE quality-of-evidence assessment of studies comparing laxatives with placebo in patients undergoing abdominal surgery

| **Certainty assessment** | | | | | | | | **Summary of findings** | |
| --- | --- | --- | --- | --- | --- | --- | --- | --- | --- |
| **Outcomes** | **No. of participants**  **(Studies)** | **Risk of bias** | **Inconsistency** | **Indirectness** | **Imprecision** | **Publication bias** | **Certainty of the evidence**  **(GRADE)** | **Anticipated absolute effects** | |
|  |  |  |  |  |  |  |  | **Risk with placebo** | **Risk difference with laxatives** |
| Time taken to pass stool (TTS)  assessed with:  Number of days Scale: 0 to 8 (days) | 416 (5 RCTs) | not serious | very serious ^a^ | not serious | not serious | none | ⨁⨁◯◯ LOW | Mean TTS  **1.57-3.1 (days)** | MD **0.83 lower** (1.39 lower to 0.26 lower) |
| Time taken to pass flatus (TTF) assessed with: Number of days Scale: 0 to 8 (days) | 330 (3 RCTs) | not serious | very serious ^b^ | not serious | serious ^c^ | none | ⨁◯◯◯ VERY LOW | Mean TTF **1.08-2.63 (days)** | MD **0.17 lower** (0.59 lower to 0.25 higher) |
| Time taken to tolerate diet (TTD) assessed with: Number of days Scale: 0 to 8 (days) | 288 (3 RCTs) | not serious | not serious | not serious | very serious ^c^ | none | ⨁⨁◯◯ LOW | Mean TTD **0.75-5.4 (days)** | MD **0.01 lower** (0.12 lower to 0.1 higher) |
| Length of hospital stay  (LOS) assessed with:  Number of days Scale: 0 to 20 (days) | 416 (5 RCTs) | not serious | serious ^d^ | not serious | very serious ^c^ | none | ⨁◯◯◯ VERY LOW | Mean LOS **1-16 (days)** | MD **0.01 higher** (1.36 lower to 1.38 higher) |

**CI:** Confidence interval; **MD:** Mean difference

#### Explanations

a - I^2^ is 94%; b - I^2^ is 87%; c - Confidence intervals included potential for no effect; d - I^2^ is 92%

**Fig. S1** Overall risk-of bias assessment for the included RCTs


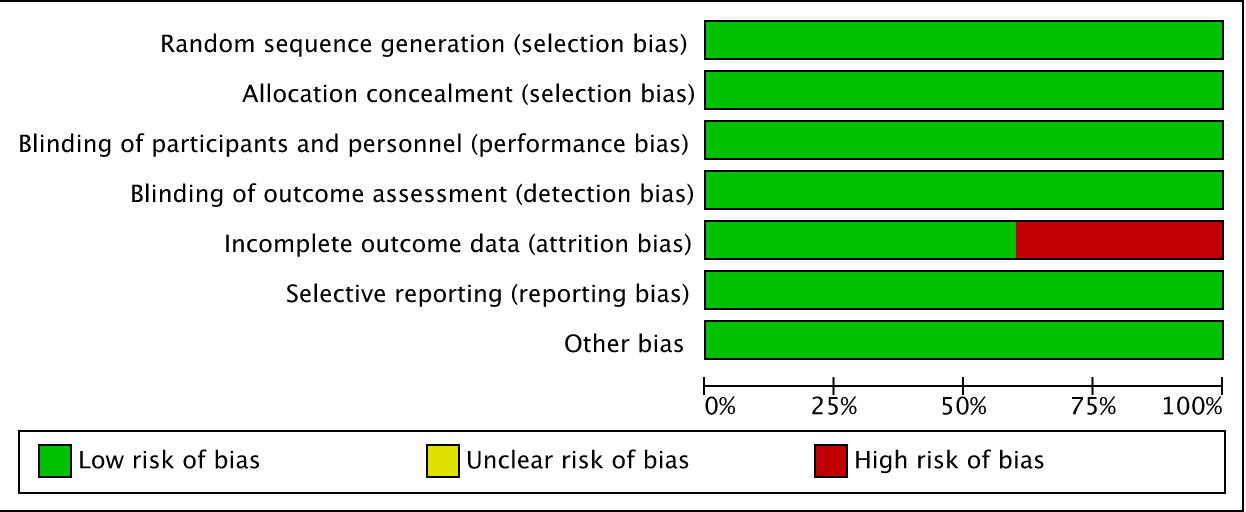


**Fig. S2** Risk-of-bias assessment for the individual studies


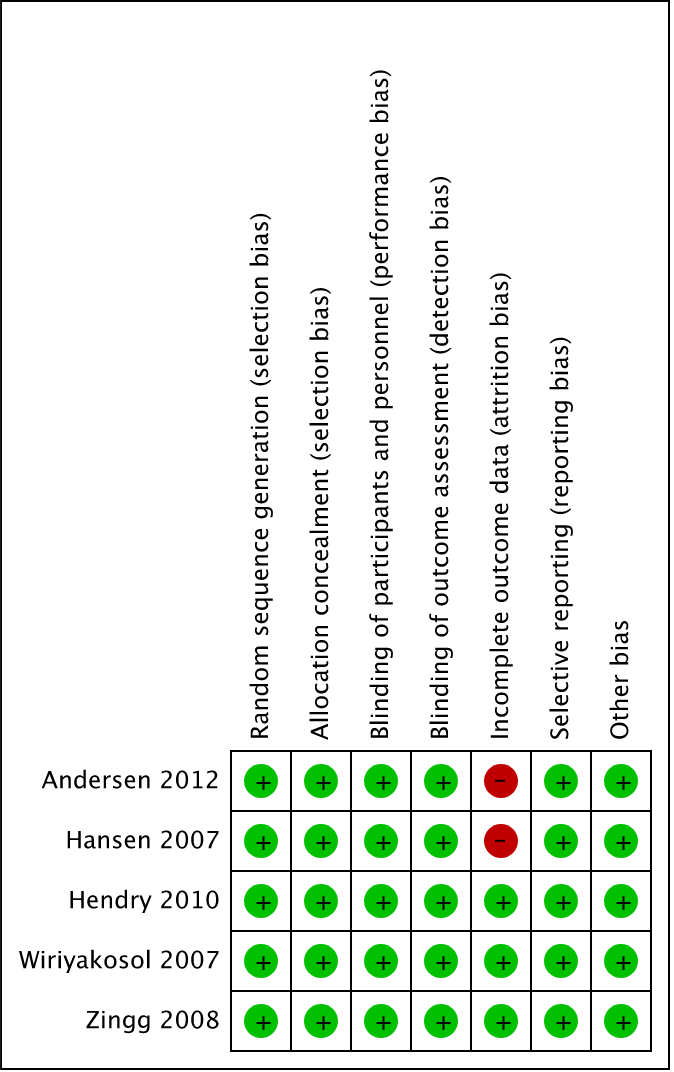

Supplement: Supplementary file 1 — Appendix S1: Supporting information [file BJS5-4-577-s001.docx]
